# The Story of the Insane from Year to Year

**Published:** 1897-04-03

**Authors:** 


### THE STORY OF THE INSANE FROM YEAR TO YEAR.

Ninth Series.

### HOSPITAL FOR THE INSANE AT ELGIN, ILLINOIS.

This is a biennial report; and during the two years the numbers increased from 1,107 to 1,178. There were 681 admissions, 106 re-admissions; 349 recoveries and 154 deaths. The percentage of recoveries calculated in the usual way is 44·47, and that of the deaths is 13·75. Intemperance in drink is put down as the cause of insanity in 46 of the admissions and hereditary tendency in 20 only; but as 366 are of unknown cause, the table is comparatively useless. The percentages of recoveries has risen considerably in this asylum during the last two years, and Dr. Loewy thinks this is owing to the adoption of the "open-door system"; to the increased proportion of attendants (implying more care and watchfulness) and to scientific treatment. No doubt all these are admirable and highly desirable things, and each may be expected to contribute to the recovery rate, but Dr. Loewy will probably find that the chief factor in the recovery rate will be the raw material he gets to work on. We are glad to learn that the amount of restraint has been reduced, and is now limited to urgent cases. The weekly cost of maintenance is about 10s. 6d. per head.

### THE WINSON GREEN ASYLUM, BIRMINGHAM.

Here the numbers fell from 634 to 619. There were 311 admissions, 82 re-admissions, 157 recoveries, 20 discharged relieved, and 107 deaths. Calculated in the usual way, the recoveries stand at 41·9 per cent., and the deaths at 17·5. Of the deaths, 36 were caused by general paralysis and 32 by other brain diseases, thus accounting for the high death-rate. There were 10 deaths from consumption and 10 from influenza. Intemperance in drink was the cause of insanity in 46 of the admissions, and hereditary influence in 36. Mr. Whitcomb discusses carefully the question of the increase of insanity, and he arrives at the conclusion that there is a slight increase of late years, and he rightly says that insanity is a disease having many causes for its production; and he adds "that considerable factors in accounting for this increase are the greater excitement under which we live and the increasing competition for the means of existence." A serious outbreak of influenza took place during the year, and, as already said, 10 patients died of it. There are about 20 private patients in this asylum, and these pay from 10s. 6d. to one guinea a week. The rate for paupers is 8s. 6d.

